# The Effectiveness of Publicly Available Web-Based Interventions in Promoting Health App Use, Digital Health Literacy, and Media Literacy: Pre-Post Evaluation Study

**DOI:** 10.2196/46336

**Published:** 2023-12-04

**Authors:** Lars König, Ralf Suhr

**Affiliations:** 1 Institut für Medizinische Soziologie und Rehabilitationswissenschaft, Charité – Universitätsmedizin Berlin Berlin Germany; 2 Stiftung Gesundheitswissen Berlin Germany

**Keywords:** digital health literacy, e-learning, health apps, health education, health literacy, media literacy, mHealth, mobile health, serious games, user experience, web-based intervention

## Abstract

**Background:**

According to the World Health Organization, implementing mobile health (mHealth) technologies can increase access to quality health services worldwide. mHealth apps for smartphones, also known as health apps, are a central component of mHealth, and they are already used in diverse medical contexts. To benefit from health apps, potential users need specific skills that enable them to use such apps in a responsible and constructive manner.

**Objective:**

This study aimed to evaluate the effectiveness of the free and widely used web-based intervention, *The APPocalypse?*. Besides providing knowledge about health apps, the web-based intervention was designed to promote digital health and media literacy by teaching skills that enable users to distinguish between trustworthy and less trustworthy health apps. It was hypothesized that after completing the web-based intervention, participants’ knowledge in the domain of health apps, their digital health literacy, and their media literacy would be higher than it was before completing the web-based intervention.

**Methods:**

The study was divided into 3 parts. During part 1, participants (n=365; 181 female, 181 male, and 3 diverse; mean age 17.74, SD 1.391 years) provided demographic information and answered the pre- and postmeasurements. The measurements included questionnaires about participants’ knowledge in the domain of health apps, digital health literacy, and media literacy. During part 2, participants had 1 week to complete the web-based intervention. During part 3, participants answered the pre- and postmeasurements again. Furthermore, they answered educational quality and user experience questionnaires. Bayesian paired samples 2-tailed *t* tests were conducted to test the hypotheses.

**Results:**

Overall, the results support the hypotheses. After completing the web-based intervention, participants demonstrated more elaborate knowledge in the domain of health apps. Specifically, they displayed higher competencies in the domains of subjective (Bayes factor_10_ [BF_10_]=1.475×10^79^; effect size δ=–1.327) and objective health app knowledge (BF_10_=8.162×10^80^; effect size δ=–1.350). Furthermore, participants demonstrated higher digital health literacy. Specifically, they displayed higher competencies in the domains of information appraisal (BF_10_=3.413×10^43^; effect size δ=–0.870), information searching (BF_10_=3.324×10^23^; effect size δ=–0.604), evaluating reliability (BF_10_=3.081×10^35^; effect size δ=–0.766), and determining relevance (BF_10_=3.451×10^24^; effect size δ=–0.618). Regarding media literacy, the results were mixed. Participants displayed higher competencies in the domain of technology literacy beliefs (BF_10_=1.533×10^21^; effect size δ=–0.570). In the domain of technology control beliefs, their competencies did not seem to improve (BF_10_=0.109; effect size δ=–0.058). In comparison to relevant benchmarks, the web-based intervention offers exceptional educational quality and a superior user experience.

**Conclusions:**

The free web-based intervention *The APPocalypse?* might promote the constructive use of health apps, digital health literacy, and media literacy. Therefore, it may contribute to achieving the health-related United Nations Sustainable Development Goals.

## Introduction

Mobile health (mHealth) describes medical and public health practices that are “supported by mobile devices, such as mobile phones, patient monitoring devices, personal digital assistants, and other wireless devices” [1]. According to the World Health Organization (WHO), the implementation and use of mHealth technologies have the potential to increase access to quality health services worldwide [2]. Furthermore, implementing mHealth technologies may contribute to achieving the health-related Sustainable Development Goals that were adopted by the United Nations [2,3].

mHealth apps for smartphones, also known as health apps, are a central component of mHealth, and they are already used in diverse medical contexts [4-9]. They are, for example, used in suicide prevention [4], the management of chronic respiratory diseases [5], cardiac rehabilitation in older adults [6], and the self-management of hypertension [7]. Various studies have shown that health apps can promote both the physical [8] and mental health [9] of their users. Despite the medical benefits of health apps, laypeople and medical professionals often find it difficult to choose specific health apps because the market offers a wide range of health apps and their quality varies greatly [10]. In addition, many health apps seem to attach too little importance to the data security and privacy of their users, which seems particularly problematic with regard to sensitive health data [11,12].

To benefit from health apps, potential users need specific skills that enable them to use health apps in a responsible and constructive manner. In an attempt to promote such skills, stakeholders from the health care sector have started to develop various educational resources [11,13-16]. A renowned German university, for example, has partnered with a federal ministry to provide young [16] and old people [15] with general information about health apps. Furthermore, the German Medical Association and the National Association of Statutory Health Insurance physicians have developed a patient information leaflet that summarizes critical questions that users should ask themselves before using health apps [13]. From a pedagogical and didactic perspective, however, many of these educational resources are not ideally designed, even though they offer valuable information.

One exception is the web-based intervention *The APPocalypse?*, which has been developed by the independent, nonprofit foundation Stiftung Gesundheitswissen [17]. The web-based intervention is superior to many other educational resources because it makes use of diverse evidence-based e-learning design principles [18]. The web-based intervention is free of charge and publicly available at the e-learning platform Gesundweiser [19], which has already won a renowned educational media award for providing outstanding educational resources [20]. The web-based intervention was developed by a multiprofessional team with the intent of teaching the necessary skills to assess the opportunities and risks of health apps. The learning content was based on guidelines and recommendations provided by various stakeholders from the health care sector, as well as scientific checklists for the evaluation of health apps [11,13,14]. Besides providing knowledge about health apps, the web-based intervention was designed to promote digital health and media literacy by teaching skills that enable users to distinguish between trustworthy and less trustworthy health apps and health information in general [21-24]. Whether a health app is trustworthy might be assessed by answering questions like, “Is the content up-to-date and reliable?”, “Is the app recommended by a health insurance company?” and “What personal data are stored?” [13]. The e-learning platform Gesundweiser was developed in Germany and is currently available in German. To enhance privacy protection, the developers decided to keep tracking to a minimum. Therefore, users do not need to provide demographic information like age and country of residence to access the platform.

The aim of this study was to evaluate the effectiveness of the free and publicly available web-based intervention with participants between the ages of 16 and 21 years by testing the following hypotheses:

- Hypothesis 1: after completing the web-based intervention, participants’ knowledge in the domain of health apps will be higher than it was before completing the web-based intervention.
- Hypothesis 2: after completing the web-based intervention, participants’ digital health literacy levels will be higher than they were before completing the web-based intervention.
- Hypothesis 3: after completing the web-based intervention, participants’ media literacy levels will be higher than they were before completing the web-based intervention.

Furthermore, the educational quality and user experience will be assessed and compared against relevant benchmarks to objectively evaluate the practical value of the web-based intervention.

## Methods

### Ethical Considerations

Before data collection, a detailed study protocol that included information about the procedures, measures, and statistical analyses was submitted to the ethics committee of the Berlin Medical Association. The ethics committee consisted of 2 medical doctors, 1 lawyer, 1 statistician, and 1 layperson. The ethics committee had no ethical or professional objections to the study protocol (reference number Eth-53/22). All participants gave their informed consent to take part in the study. At the end of the study, participants received a €50 (US $54.67) internet-based shop voucher as compensation for participating in the study.

### Target Population

Research has found that young people seem to be the main users of health apps [25,26]. Furthermore, the WHO stresses that competencies in the domain of health technology and digital health literacy should be taught to school-age children [27,28]. Moreover, data show that young people especially seem eager to participate in web-based learning activities [29]. Additionally, to comply with the German General Data Protection Regulation (Artikel 8, Datenschutz-Grundverordnung), participants had to be at least 16 years of age. Therefore, students between the ages of 16 and 21 years were recruited as participants. A professional market research institute was responsible for the recruitment process. To increase the representativeness of the sample, participants were recruited from all German states, from the most common types of schools, and from different grades.

### Power Analysis

Before data collection, an a priori power analysis was calculated with the statistical software G*Power (University of Düsseldorf) to determine an adequate sample size [30]. Detailed information about the power analysis is shown in Table 1. Results indicated that at least 327 participants were needed to identify a small effect with sufficient power.

**Table 1:** Protocol of the power analysis.

Parameter			Value
**General information**			
	*t* tests	Means: difference between 2 dependent means (matched pairs)	
	Analysis	A priori: compute required sample size	
**Input**			
	Tails	2	
	Effect size dz	0.2	
	α error probability	0.05	
	Power (1-β error probability)	0.95	
**Output**			
	Noncentrality parameter δ	3.6166283	
	Critical *t*	1.9672675	
	*df*	326	
	Total sample size	327	
	Actual power	0.9501171	

### Procedure

The pre- and postmeasurement study was conducted on the internet. Data collection took place in December 2022 and was conducted by the market research institute SPLENDID RESEARCH GmbH (Hamburg, Germany). Participants were drawn from a web-based research panel and invited through email to take part in the study. If needed, individuals were reminded through email to participate in the 3 different stages of the study. Before the study started, participants were provided with detailed information about the general purpose of the study, the upcoming questionnaires, and data security measures. Before data collection, all participants gave their informed consent to take part in the study. The study was divided into 3 parts. During part 1, participants provided demographic information and answered the pre- and postmeasurements. The pre- and postmeasurements included questionnaires about participants’ knowledge in the domain of health apps, digital health literacy, and media literacy. During part 2, participants had 1 week to complete the web-based intervention. During part 3, participants answered the pre- and postmeasurements again. Furthermore, they answered educational quality and user experience questionnaires. Research has shown that an adequate compensation for participating in research studies might improve data quality [31]. Therefore, at the end of the study, participants received a €50 (US $54.67) internet-based shop voucher as compensation for participating in the study.

### Material

**Figure 1:**
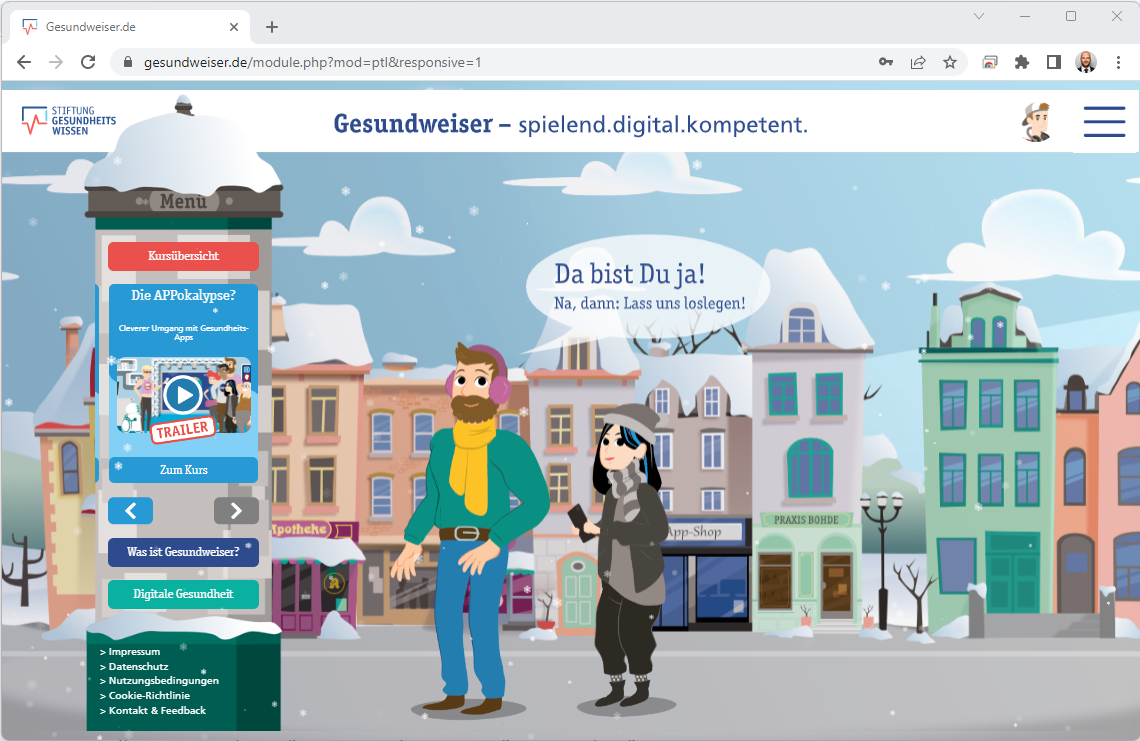
Landing page of the e-learning platform Gesundweiser.

The web-based intervention was designed by a multiprofessional team to teach the necessary skills to assess the opportunities and risks of health apps. The learning content was based on guidelines and recommendations provided by various stakeholders from the health care sector, as well as scientific checklists for the evaluation of health apps [11,13,14]. Besides providing knowledge about health apps, the web-based intervention was designed to promote digital health and media literacy by teaching skills that enable users to distinguish between trustworthy and less trustworthy health apps and health information in general [21-24]. The web-based intervention consists of 5 mandatory modules, 1 optional module, and 1 final test. Within the modules, animated videos, instructional texts, and diverse interactive tasks are used to create a motivational learning environment. The entire web-based intervention can be completed in about 90 minutes. The web-based intervention is called *The APPocalypse?* and can be accessed freely through the e-learning platform Gesundweiser. Figure 1 [19] shows the landing page of the e-learning platform. Textbox 1 provides an overview of the learning content of the web-based intervention.

**Overview of the learning content of the web-based intervention. Modules and their content and key messages are given.**

**Mandatory module 1**

- Health apps are becoming increasingly popular. However, there are a few things you should keep in mind when using them:
  There are many different types of health apps.
  Health apps can help people stay healthy or better manage an illness.
  Health apps can also pose risks, eg, if they pass on incorrect information.
  The risks of a health app are not always apparent at first glance.

**Mandatory module 2**

- To find a trustworthy health app that is right for you, there are a few things you can consider even before you download the app:
  Ratings and download numbers are only indications of an app’s popularity and visibility.
  The app description should contain as much concrete information as possible about the purpose of the app.
  A good app should make it clear who it is made for.
  Seals and certificates only provide initial indications of the trustworthiness of an app.

**Mandatory module 3**

- If you want to use a health app, then it makes sense to pay attention to a few things:
  Already in the app store or, at the latest, in the imprint, it should be clearly recognizable who the provider of the app is.
  Can you see how the app is funded? In this way, you can deduce the possible interests of the producer.
  It is advisable to check in advance which additional functions have to be purchased. This way, you can avoid high costs.
  Advertising within the app should be marked as such, eg, by the note “advertisement.”

**Mandatory module 4**

- Health apps and protecting your data—what can you look out for when using apps?
  Health data must be particularly protected because it tells others something about your health.
  The privacy statement states what data an app collects and whether it is shared.
  The permissions an app asks for should match the functions of the app.
  You can also change an app’s access rights on your smartphone later.

**Mandatory module 5**

- How can you tell whether a health app is of good quality?
  The texts are neutrally worded and up-to-date, and the recommendations are clear.
  Authors are indicated, and their qualifications match the content.
  The illustrations in the app are labeled in a meaningful way and offer explanatory notes.
  Individual settings can be made so that the app delivers the right functionality for me.

**Optional module**

- The optional module provides information about health apps that are paid for by health insurance companies.

**Final test**

- The learning content is tested in the final test.

### Measures

#### Health App Knowledge

Currently, no validated instrument exists that assesses the specific health app knowledge of the web-based intervention *The APPocalypse?*. Therefore, based on the content of the web-based intervention, a questionnaire was developed to assess subjective health app knowledge. Participants rated 4 items (eg, “I can explain the characteristics of a trustworthy health app”) on scales ranging from 1 (totally disagree) to 6 (totally agree). A total score was generated by calculating the mean. Additionally, a multiple-choice test was developed to assess objective health app knowledge. The test was based on the content of the web-based intervention and consisted of 11 multiple-choice questions with 4 response options each. For example, 1 question asked, “Which of these applications are health apps?” The following were the response options: (1) “pedometer application,” (2) “application for better sleep,” (3) “vaccination calendar application,” and (4) “food diary application.” For every question, participants received 1 point if they chose all the correct response options. A total score was generated by summing up all the points. The health app knowledge questionnaire and the multiple-choice test can be found in Multimedia Appendix 1.

#### Digital Health Literacy

The subscale “information appraisal” from the eHealth Literacy Scale was adapted to measure digital health literacy in the context of health apps [21,24]. The subscale consisted of 4 items (eg, “I can distinguish between trustworthy and questionable health apps”) that were rated on scales ranging from 1 (strongly disagree) to 5 (strongly agree). Furthermore, 3 of the 7 subscales from the Digital Health Literacy Instrument were adapted to measure digital health literacy in the context of health apps [22,32]. The subscales “information searching” (eg, “When you search the internet for information on health apps, how easy or difficult is it for you to find the exact information you are looking for?”), “evaluating reliability” (eg, “When you search the internet for information on health apps, how easy or difficult is it for you to decide whether the information is reliable or not?”), and “determining relevance” (eg, “When you search the internet for information on health apps, how easy or difficult is it for you to decide if the information you found is applicable to you?”) consisted of 3 items each and were rated on scales ranging from 1 (very difficult) to 4 (very easy). The described subscales were chosen because they focus on the specific skills that were addressed within the web-based intervention. A total score was generated for every subscale by calculating the mean.

#### Media Literacy

A total of 2 of the 3 subscales from the Technology Commitment Scale were adapted to measure media literacy in the context of health apps [33]. The subscales “technology literacy beliefs” (eg, “When dealing with health apps, I am often afraid of failing,” reverse coded) and “technology control beliefs” (eg, “Whether I am successful in using health apps depends essentially on me”) consisted of 4 items each and were rated on scales ranging from 1 (strongly disagree) to 5 (strongly agree). A total score was generated for every subscale by calculating the mean.

#### Educational Quality

The subscale “learning and value” from the Student Evaluation of Educational Quality instrument was adapted to measure educational quality [34,35]. The subscale consisted of 5 items (eg, “My interest in the subject has increased as a consequence of this online course”) that were rated on scales ranging from 1 (strongly disagree) to 5 (strongly agree). A total score was generated by calculating the mean. In a large study that evaluated 3660 learning courses, the average learning and value score was 4.0 [35]. Therefore, a learning and value score of 4.0 or higher will serve as a threshold that indicates superior educational quality. No hypotheses were made about educational quality. Therefore, the analysis is exploratory.

#### User Experience

The 4 subscales from the Visual Aesthetics of Website Inventory were used to measure user experience [36]. The subscales “simplicity” (eg, “The layout appears well structured”) and “diversity” (eg, “The layout appears dynamic”) consisted of 5 items each. The subscales “colorfulness” (eg, “The color composition is attractive”) and “craftsmanship” (eg, “The layout appears professionally designed”) consisted of 4 items each. All items were rated on scales ranging from 1 (strongly disagree) to 7 (strongly agree). A total score was generated for every subscale by calculating the mean. Furthermore, an overall score was generated by calculating the overall mean. Detailed information about the subfacets of user experience and their theoretical and practical importance can be found elsewhere [36]. Previous research has shown that an overall score of 4.5 or higher is associated with a generally good impression of websites [37]. Moreover, a benchmark study found that the average e-learning platform does not manage to surpass this threshold [38]. Therefore, an overall score of 4.5 or higher will serve as a threshold that indicates a superior user experience. No hypotheses were made about user experience. Therefore, the analyses are exploratory.

### Statistical Analyses

Statistical analyses were conducted with the statistical software SPSS (version 29.0.0.0; IBM Corp) [39] and JASP (version 0.16.4; University of Amsterdam) [40]. Cronbach α was calculated with SPSS for every scale to ensure the quality of the measures. Bayesian paired samples 2-tailed *t* tests were conducted with JASP to test the hypotheses. Table 2 shows a detailed protocol of the Bayesian analyses with all the needed information to replicate the analyses. Given the study design, Bayes factors (BFs) might be interpreted as anecdotal evidence (BF_10_: 1-3), moderate evidence (BF_10_: 3-10), strong evidence (BF_10_: 10-30), very strong evidence (BF_10_: 30-100), or extreme evidence (BF_10_: >100) for the alternative hypothesis compared to the null hypothesis considering the observed data [41]. For all Bayesian analyses, detailed information about the robustness of the calculated Bayes factors and the corresponding effect sizes will be reported. Readers not familiar with Bayesian statistical analyses in medical contexts and the statistical software JASP can find introductory material elsewhere [41-43].

**Table 2:** Protocol of Bayesian analysis.

Parameter			Value	
**General information**				
	*t* tests	Bayesian paired samples *t* test		
**Input**				
	Alternative hypothesis	Measure 1 ≠ measure 2		
	Bayes factor	BF_10_		
	Tests	Student		
	Missing values	Exclude cases per dependent variable		
	Plots	(1) Prior and posterior (additional information; 95% CI); (2) Bayes factor robustness check (additional information); (3) sequential analysis		
	Additional statistics	Descriptives		
	Prior	Default (Cauchy scale 0.707)		

## Results

### Sample Characteristics

A total of 365 participants (181 female, 181 male, and 3 diverse) completed the study by finishing the mandatory modules and final test and by answering all measures. All study participants were included in data analyses. As intended, participants came from all German states, the most common types of schools, and different grades. The average participant was 18 years of age (mean 17.74, SD 1.391 years). Table 3 provides detailed information about the sample distribution by state, type of school, and grade.

**Table 3:** Sample distribution by state, type of school, and grade.

Characteristics			Values, n (%)
**State in German (state in English)**			
	Baden-Württemberg (Baden-Württemberg)	18 (4.9)	
	Bayern (Bavaria)	49 (13.4)	
	Berlin (Berlin)	20 (5.5)	
	Brandenburg (Brandenburg)	22 (6)	
	Bremen (Bremen)	8 (2.2)	
	Hamburg (Hamburg)	15 (4.1)	
	Hessen (Hesse)	36 (9.9)	
	Mecklenburg-Vorpommern (Mecklenburg-West Pomerania)	12 (3.3)	
	Niedersachsen (Lower Saxony)	38 (10.4)	
	Nordrhein-Westfalen (Northrhine-Westphalia)	43 (11.8)	
	Rheinland-Pfalz (Rhineland Palatinate)	15 (4.1)	
	Saarland (Saarland)	8 (2.2)	
	Sachsen (Saxony)	32 (8.8)	
	Sachsen-Anhalt (Saxony-Anhalt)	20 (5.5)	
	Schleswig-Holstein (Schleswig Holstein)	12 (3.3)	
	Thüringen (Thuringia)	17 (4.7)	
**Type of school**			
	Hauptschule (eg, junior high schools)	16 (4.4)	
	Realschule, Werkrealschule, Sekundarschule, or Realschule plus (eg, secondary high schools)	69 (18.9)	
	Schule mit mehreren Bildungsgängen (z.B. Gesamtschule, Gemeinschaftsschule oder Stadtteilschule; eg, comprehensive schools)	44 (12.1)	
	Gymnasium (eg, grammar schools)	129 (35.3)	
	Förder-oder Sonderschule (eg, special schools)	1 (0.3)	
	Berufliche Schule (eg, vocational schools)	103 (28.2)	
	Other	3 (0.8)	
**Grade**			
	9	26 (7.1)	
	10	89 (24.4)	
	11	66 (18.1)	
	12	85 (23.3)	
	13	40 (11)	
	Other	59 (16.2)	

### Quality of Measures

Most measures and their instructions were slightly adapted to be more applicable in the context of health apps. To ensure the quality of the measures, Cronbach α was calculated for every scale. Common conventions define values of .7 or higher as acceptable [44,45]. Every scale surpassed this commonly used threshold. Table 4 provides further information about the scales and the corresponding Cronbach α values.

**Table 4:** Number of scale items and corresponding Cronbach α value (N=365).

Measures			Cronbach α		Items, n
**Health app knowledge**					4
	Subjective knowledge (pre)	.912			
	Subjective knowledge (post)	.873			
**Digital health literacy**					
	Information appraisal (pre)	.816		4	
	Information appraisal (post)	.819		4	
	Information searching (pre)	.748		3	
	Information searching (post)	.757		3	
	Evaluating reliability (pre)	.742		3	
	Evaluating reliability (post)	.701		3	
	Determining relevance (pre)	.772		3	
	Determining relevance (post)	.791		3	
**Media literacy**					4
	Technology literacy beliefs (pre)	.904			
	Technology literacy beliefs (post)	.890			
	Technology control beliefs (pre)	.742			
	Technology control beliefs (post)	.842			
**Educational quality**					5
	Learning and value	.800			
**User experience**					
	Overall score	.924		18	
	Simplicity	.820		5	
	Diversity	.813		5	
	Colorfulness	.736		4	
	Craftsmanship	.782		4	

### Health App Knowledge

**Figure 2:**
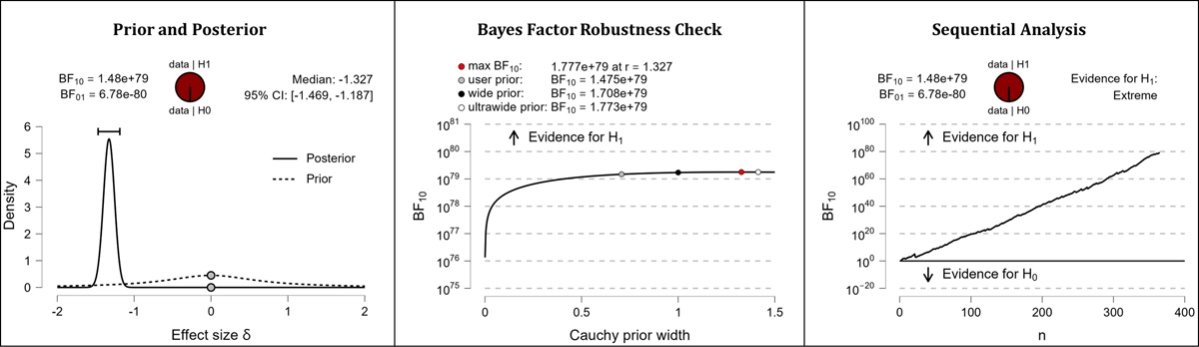
Health app knowledge: subjective knowledge. BF: Bayes factor.

**Figure 3:**
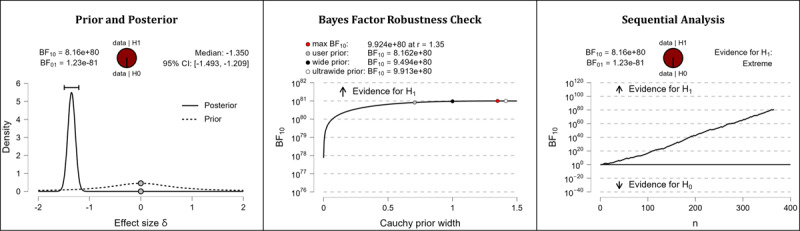
Health app knowledge: objective knowledge. BF: Bayes factor.

It was hypothesized that after completing the web-based intervention, participants’ knowledge in the domain of health apps would be higher than it was before completing the web-based intervention. The results of the Bayesian analyses show extreme support for the hypothesis. After completing the web-based intervention, participants displayed higher competencies in the domains of subjective health app knowledge (extreme evidence: BF_10_=1.475×10^79^; error %=2.129×10^–84^; effect size δ=–1.327) and objective health app knowledge (extreme evidence: BF_10_=8.162×10^80^; error %=5.117×10^–86^; effect size δ=–1.350). Table 5 shows the descriptive statistics of the corresponding pre- and postmeasurements. Figure 2 (subjective health app knowledge) and Figure 3 (objective health app knowledge) provide detailed information about the robustness of the calculated Bayes factors and the corresponding effect sizes.

**Table 5:** Descriptive statistics of the pre- and postmeasurements. Health app knowledge: subjective knowledge ranged from 1 (low) to 6 (high) and objective knowledge ranged from 0 (low) to 11 (high); digital health literacy: information appraisal ranged from 1 (low) to 5 (high), information searching ranged from 1 (low) to 4 (high), evaluating reliability ranged from 1 (low) to 4 (high), and determining relevance ranged from 1 (low) to 4 (high); and media literacy: technology literacy beliefs ranged from 1 (low) to 5 (high) and technology control beliefs ranged from 1 (low) to 5 (high).

Measures		Mean (SD)	SE	Coefficient of variation	95% CI
**Health app knowledge**					
	Subjective knowledge (pre)	3.458 (1.193)	0.062	0.345	3.335-3.581
	Subjective knowledge (post)	5.016 (0.710)	0.037	0.142	4.943-5.089
	Objective knowledge (pre)	2.748 (2.003)	0.105	0.729	2.542-2.954
	Objective knowledge (post)	5.712 (2.024)	0.106	0.354	5.504-5.921
**Digital health literacy**					
	Information appraisal (pre)	3.375 (0.784)	0.041	0.232	3.295-3.456
	Information appraisal (post)	4.103 (0.637)	0.033	0.155	4.038-4.169
	Information searching (pre)	2.511 (0.617)	0.032	0.246	2.447-2.574
	Information searching (post)	2.924 (0.561)	0.029	0.192	2.866-2.982
	Evaluating reliability (pre)	2.353 (0.638)	0.033	0.271	2.288-2.419
	Evaluating reliability (post)	2.908 (0.601)	0.031	0.207	2.846-2.970
	Determining relevance (pre)	2.429 (0.648)	0.034	0.267	2.363-2.496
	Determining relevance (post)	2.900 (0.626)	0.033	0.216	2.835-2.964
**Media literacy**					
	Technology literacy beliefs (pre)	3.462 (1.007)	0.053	0.291	3.359-3.566
	Technology literacy beliefs (post)	4.041 (0.796)	0.042	0.197	3.959-4.123
	Technology control beliefs (pre)	3.321 (0.709)	0.037	0.214	3.248-3.394
	Technology control beliefs (post)	3.379 (0.919)	0.048	0.272	3.284-3.473

### Digital Health Literacy

**Figure 4:**
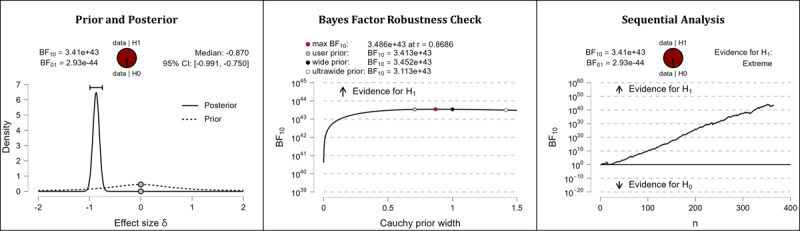
Digital health literacy: information appraisal. BF: Bayes factor.

**Figure 5:**
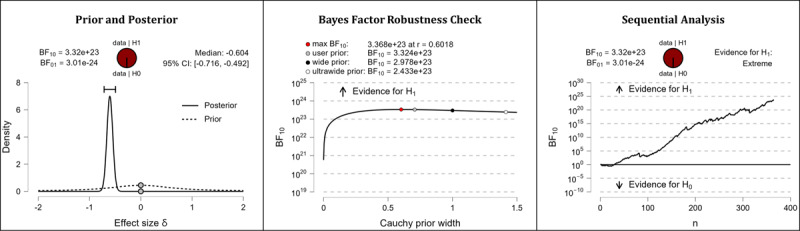
Digital health literacy: information searching. BF: Bayes factor.

**Figure 6:**
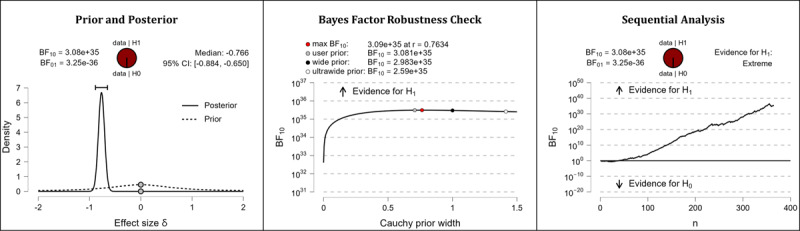
Digital health literacy: evaluating reliability. BF: Bayes factor.

**Figure 7:**
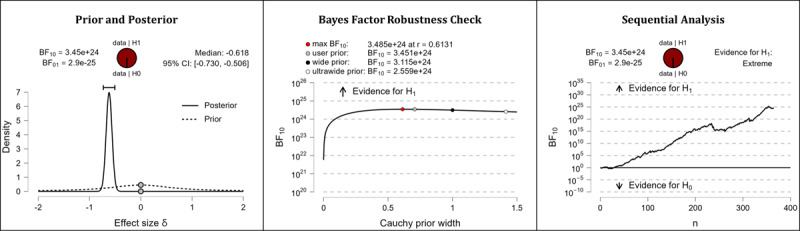
Digital health literacy: determining relevance. BF: Bayes factor.

It was hypothesized that after completing the web-based intervention, participants’ digital health literacy levels would be higher than they were before completing the web-based intervention. The results of the Bayesian analyses show extreme support for the hypothesis. After completing the web-based intervention, participants displayed higher competencies in the domains of information appraisal (extreme evidence: BF_10_=3.413×10^43^; error %=6.978×10^–46^; effect size δ=–0.870), information searching (extreme evidence: BF_10_=3.324×10^23^; error %=9.840×10^–26^; effect size δ=–0.604), evaluating reliability (extreme evidence: BF_10_=3.081×10^35^; error %=8.626×10^–39^; effect size δ=–0.766), and determining relevance (extreme evidence: BF_10_=3.451×10^24^; error %=8.938×10^–27^; effect size δ=–0.618). Table 5 shows the descriptive statistics of the corresponding pre- and postmeasurements. Figure 4 (information appraisal), Figure 5 (information searching), Figure 6 (evaluating reliability), and Figure 7 (determining relevance) provide detailed information about the robustness of the calculated Bayes factors and the corresponding effect sizes.

### Media Literacy

**Figure 8:**
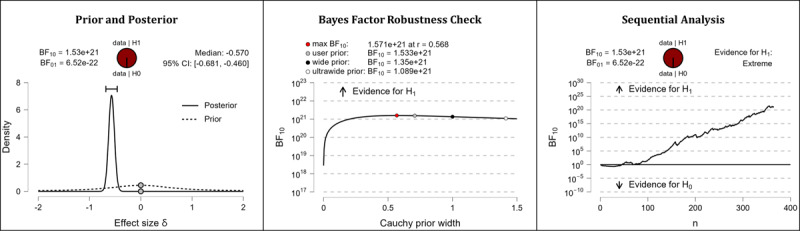
Media literacy: technology literacy beliefs. BF: Bayes factor.

**Figure 9:**
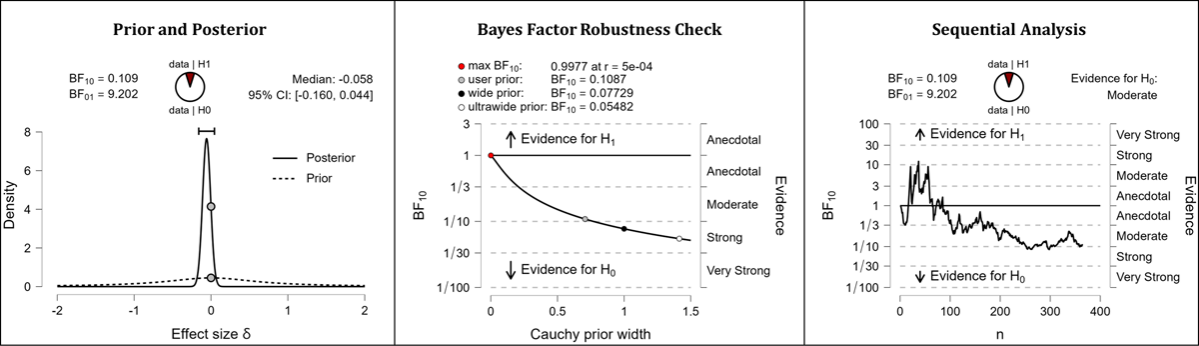
Media literacy: technology control beliefs. BF: Bayes factor.

It was hypothesized that after completing the web-based intervention, participants’ media literacy levels would be higher than they were before completing the web-based intervention. The results of the Bayesian analyses are mixed. After completing the web-based intervention, participants displayed higher competencies in the domain of technology literacy beliefs (extreme evidence: BF_10_=1.533×10^21^; error %=2.776×10^–28^; effect size δ=–0.570). In the domain of technology control beliefs, however, participants’ competencies did not seem to improve (BF_10_=0.109; error %=0.199; effect size δ=–0.058). Table 5 shows the descriptive statistics of the corresponding pre- and postmeasurements. Figure 8 (technology literacy beliefs) and Figure 9 (technology control beliefs) provide detailed information about the robustness of the calculated Bayes factors and the corresponding effect sizes.

### Educational Quality

The educational quality measure ranged from 1 (low rating) to 5 (high rating). The mean of the learning and value score (mean 4.361, SE 0.029, SD 0.549; coefficient of variation=0.126; minimum=1.600, maximum=5.000) surpassed the threshold of 4.0 which indicates a superior educational quality.

### User Experience

The user experience measures ranged from 1 (low rating) to 7 (high rating). The means of the overall (mean 5.660, SE 0.040, SD 0.761; coefficient of variation=0.134; minimum=2.944, maximum=7.000), simplicity (mean 5.689, SE 0.044, SD 0.839; coefficient of variation=0.147; minimum=2.600, maximum=7.000), diversity (mean 5.620, SE 0.045, SD 0.860; coefficient of variation=0.153; minimum=2.200, maximum=7.000), colorfulness (mean 5.628, SE 0.047, SD 0.890; coefficient of variation=0.158; minimum=2.250, maximum=7.000), and craftsmanship (mean 5.707, SE 0.048, SD 0.925; coefficient of variation=0.162; minimum=1.750, maximum=7.000) scores surpassed the threshold of 4.5, which indicates a superior user experience. The raw data set contains further variables that have not been described because they exceed the scope of this study.

## Discussion

### Principal Findings

The central aim of this study was to evaluate the effectiveness of the free and widely used web-based intervention *The APPocalypse?*. It was hypothesized that after completing the web-based intervention, participants’ knowledge in the domain of health apps (hypothesis 1), their digital health literacy (hypothesis 2), and their media literacy (hypothesis 3) would be higher than it was before completing the web-based intervention. Overall, the results of the Bayesian analyses support these hypotheses. After completing the web-based intervention, participants demonstrated more elaborate knowledge in the domain of health apps. More specifically, they demonstrated higher subjective and objective health app knowledge. Furthermore, participants demonstrated higher digital health literacy. More specifically, they demonstrated more elaborate competencies in the domains of information appraisal, information searching, evaluating reliability, and determining relevance. Regarding media literacy, the results were mixed. After completing the web-based intervention, participants demonstrated more elaborate competencies in the domain of technology literacy beliefs. However, their competencies in the domain of technology control beliefs did not seem to improve.

The mixed media literacy results need further explanation. The discrepancy might be explained by the nature of the items that were used in the questionnaires. The items used to measure technology literacy beliefs described competencies that regard specific problems (eg, “When dealing with health apps, I am often afraid of failing”). Such problems can be addressed in web-based interventions through specific training practices that improve the needed competencies and thereby reduce associated fears. The items used to measure technology control beliefs, however, did not address specific problems. Instead, they described more general beliefs (eg, “Whether I am successful in using health apps depends essentially on me”), which are harder to address in training practices. Furthermore, the wording of the technology control belief items resembles formulations that are typically used to describe and measure psychological personality traits, which are known to be relatively stable over time and hard to change [46,47].

Another central aim of this study was to objectively evaluate the practical value of the web-based intervention. To this end, the educational quality and user experience of the web-based intervention were assessed and compared against relevant benchmarks and thresholds. The results show that the web-based intervention offers exceptional educational quality and a superior user experience. These results are especially relevant because educational quality and user experience might influence users’ willingness to participate in web-based interventions and improve their knowledge acquisition [48-51]. In the future, the effectiveness and user experience of the web-based intervention might be further improved by implementing virtual reality components and opportunities for collaboration [52,53]. Moreover, the current results might be used to identify skill sets that could be addressed in more detail when updating the learning content of the web-based intervention. This might be especially relevant in regard to the technology control beliefs, which did not seem to improve substantially. Previous research suggests that even psychological traits might change over time, and in some circumstances, an updated version of the web-based intervention could benefit from additional tasks that especially address technology control beliefs [54].

### Limitations and Future Directions

Even though the results of this study suggest that web-based interventions might promote digital health literacy, media literacy, and the constructive use of health apps, there are limitations to the generalizability of the results, which arise from certain characteristics of the study. Especially significant are 2 of these limitations.

The first limitation concerns the chosen study sample. Students between the ages of 16 and 21 years were recruited because young people are the main users of health apps [25,26], and the WHO stresses the importance of teaching digital health literacy skills to school-age children [27,28]. However, one might argue that health apps can be especially beneficial for older adults, who are often confronted with diverse health problems [55,56]. Furthermore, older people often possess limited skills in the domains of digital health literacy and media literacy [57,58]. Following this argumentation, older people might be in special need of web-based interventions that promote their digital health literacy, media literacy, and their constructive use of health apps. Therefore, future studies should replicate this study with more diverse age groups to explore whether the current findings are generalizable to other age groups.

The second limitation concerns the study’s design. To test the hypotheses, this study adopted a pre- and postmeasurement design that assessed the competencies of the same participants before and after completing the web-based intervention. Such research designs are widely used in educational and medical contexts and offer various advantages (eg, economic implementation) [59]. These research designs, however, have 2 major drawbacks. First, pre- and postmeasurement designs do not allow for causal inferences because they do not follow a strict experimental protocol [60,61]. Second, pre- and postmeasurement designs are at risk of inducing demand effects [62-64]. After answering the premeasurements, for example, participants might have guessed that the web-based intervention is designed to improve digital health and media literacy skills and therefore adjusted their answers in the postmeasurements accordingly. It needs to be stressed, however, that such adjustments are relatively unlikely regarding the objective knowledge acquisition test that was administered. Nevertheless, future studies should replicate this study within a rigorous experimental between-subjects design to allow causal inferences and avoid demand effects.

### Conclusions

Overall, the free and widely used web-based intervention *The APPocalypse?* might promote the constructive use of health apps, digital health literacy, and media literacy. Therefore, it might help to reach the health-related Sustainable Development Goals that were adopted by the United Nations [2,3]. Furthermore, because the web-based intervention offers exceptional educational quality and a superior user experience, it might motivate users to complete the web-based intervention, thereby sustainably promoting their skills in the domains of digital health and health apps [48-51].

## Appendix

Multimedia Appendix 1 Investigator-developed data collection instruments.

## Abbreviations

BF: Bayes factor
mHealth: mobile health
WHO: World Health Organization

## Data Availability

The data sets generated and analyzed during this study are available from the Stiftung Gesundheitswissen on reasonable request.
