# Supplementary material for: The Effectiveness of Publicly Available Web-Based Interventions in Promoting Health App Use, Digital Health Literacy, and Media Literacy: Pre-Post Evaluation Study
Source: J Med Internet Res. 2023 Dec 4;25:e46336. doi: 10.2196/46336 (PMC10728793; doi:10.2196/46336)
Supplement: Multimedia Appendix 1 [file jmir_v25i1e46336_app1.docx]

# Investigator-developed data collection instruments

Table of contents

[Investigator-developed data collection instruments 1](#_Toc148199561)

[1.1 KS – Kursspezifische Selbsteinschätzung 2](#_Toc148199562)

[1.2 KW – Kursspezifischer Wissenstest 3](#_Toc148199563)

## KS – Kursspezifische Selbsteinschätzung

**Instruktion**

| **Instruktion** |
| --- |
| Inwieweit stimmen Sie folgenden Aussagen zu?  Bitte beantworten Sie alle Fragen, auch wenn Sie noch keine Gesundheits-App genutzt haben. Wählen Sie in diesem Fall die am ehesten auf Sie zutreffende Antwort. |

**Items**

| **Nr.** | **Variablenname** | **Item** |
| --- | --- | --- |
| 1 | KS1 | Ich kann erklären, was eine Gesundheits-App ausmacht. |
| 2 | KS2 | Ich kann erklären, welche Merkmale für eine vertrauenswürdige Gesundheits-App sprechen. |
| 3 | KS3 | Ich kann erklären, worauf man vor dem Download einer Gesundheits-App achten sollte. |
| 4 | KS4 | Ich kann erklären, warum der Datenschutz bei Gesundheits-Apps eine besondere Rolle spielt. |

**Antwortoptionen**

| **Codierung** | **Antwortoption** |
| --- | --- |
| 1 | stimme überhaupt nicht zu |
| 2 | stimme nicht zu |
| 3 | stimme eher nicht zu |
| 4 | stimme eher zu |
| 5 | stimme zu |
| 6 | stimme voll und ganz zu |

## KW – Kursspezifischer Wissenstest

**Instruktion**

| **Instruktion** |
| --- |
| Was zeichnet Gesundheits-Apps aus? |

**Items**

| **Nr.** | **Variablenname** | **Item** |
| --- | --- | --- |
| 1 | KW01A | Sie können unterstützen gesund zu leben. |
| 2 | KW01B | Sie können bedenkenlos genutzt werden. |
| 3 | KW01C | Sie können helfen mit Krankheiten besser umzugehen. |
| 4 | KW01D | Sie sind als digitale Anwendungen verfügbar. |

**Instruktion**

| **Instruktion** |
| --- |
| Welche dieser Apps sind Gesundheits-Apps? |

**Items**

| **Nr.** | **Variablenname** | **Item** |
| --- | --- | --- |
| 1 | KW02A | Schrittzähler-App |
| 2 | KW02B | App für besseren Schlaf |
| 3 | KW02C | Impfkalender-App |
| 4 | KW02D | Ernährungstagebuch-App |

**Instruktion**

| **Instruktion** |
| --- |
| Wie findest du schon vor dem App-Download heraus, welche Apps von guter Qualität sind? |

**Items**

| **Nr.** | **Variablenname** | **Item** |
| --- | --- | --- |
| 1 | KW03A | Je mehr Downloadzahlen, desto besser die App. |
| 2 | KW03B | In den App-Beschreibungen werden Zweck und Grenzen der App angegeben. |
| 3 | KW03C | Die Sterne-Bewertungen geben die Qualität an. |
| 4 | KW03D | Einen Qualitäts-Check lässt sich erst nach dem Download durchführen. |

**Instruktion**

| **Instruktion** |
| --- |
| Was bedeutet die Zeichen „USK ab 6 freigegeben“? |

**Items**

| **Nr.** | **Variablenname** | **Item** |
| --- | --- | --- |
| 1 | KW04A | Die App wurde für Menschen ab 6 Jahren entwickelt. |
| 2 | KW04B | Das USK-Zeichen gibt das Mindestalter an, für das die App unbedenklich ist. |
| 3 | KW04C | USK-Zeichen gibt es nicht für Apps. |
| 4 | KW04D | Das USK-Zeichen gibt an, dass die App für Menschen ab 6 Jahren empfohlen ist. |

**Instruktion**

| **Instruktion** |
| --- |
| Gesundheitsdaten geben Auskunft über deine körperliche und geistige Gesundheit. Welche dieser Angaben sind Gesundheitsdaten? |

**Items**

| **Nr.** | **Variablenname** | **Item** |
| --- | --- | --- |
| 1 | KW05A | Körpergröße |
| 2 | KW05B | Impfungen |
| 3 | KW05C | Alter |
| 4 | KW05D | Puls |

**Instruktion**

| **Instruktion** |
| --- |
| Was dürfen App-Anbieter mit deinen Gesundheits-Daten machen? |

**Items**

| **Nr.** | **Variablenname** | **Item** |
| --- | --- | --- |
| 1 | KW06A | App-Anbieter müssen offen mitteilen, welche Daten sie erheben. |
| 2 | KW06B | App-Anbieter dürfen die Daten ohne Weiteres an Dritte weitergeben. |
| 3 | KW06C | App-Anbieter müssen die Daten löschen, wenn du sie dazu aufforderst. |
| 4 | KW06D | App-Anbieter dürfen ohne weitere Zustimmung die Daten zu Forschungswecken nutzen. |

**Instruktion**

| **Instruktion** |
| --- |
| Was trifft auf das Impressum zu? |

**Items**

| **Nr.** | **Variablenname** | **Item** |
| --- | --- | --- |
| 1 | KW07A | Was es enthalten muss, regelt ein spezielles Gesetz. |
| 2 | KW07B | Im Impressum steht, wer für die App verantwortlich ist. |
| 3 | KW07C | Alle Plattformen, sowohl private als auch geschäftliche, benötigen ein Impressum. |
| 4 | KW07D | Ein Impressum enthält immer Name und Anschrift des Anbieters. |

**Instruktion**

| **Instruktion** |
| --- |
| Was trifft auf Informationen von guter Qualität zu? |

**Items**

| **Nr.** | **Variablenname** | **Item** |
| --- | --- | --- |
| 1 | KW08A | Sie sind neutral formuliert. |
| 2 | KW08B | Sie sind aktuell. |
| 3 | KW08C | Sie sind leicht auffindbar. |
| 4 | KW08D | Sie basieren auf seriösen Quellen. |

**Instruktion**

| **Instruktion** |
| --- |
| Wie können sich kostenfreie Gesundheits-Apps finanzieren? |

**Items**

| **Nr.** | **Variablenname** | **Item** |
| --- | --- | --- |
| 1 | KW09A | In-App-Käufe |
| 2 | KW09B | Datenverkauf |
| 3 | KW09C | Sponsoren |
| 4 | KW09D | Werbung |

**Instruktion**

| **Instruktion** |
| --- |
| Welche Kriterien sollten gute Apps erfüllen? |

**Items**

| **Nr.** | **Variablenname** | **Item** |
| --- | --- | --- |
| 1 | KW10A | Informationen werden in Fachsprache vermittelt. |
| 2 | KW10B | Apps enthalten Videos statt Text. |
| 3 | KW10C | Sie liefern auf deine Eingaben angepasste Ergebnisse. |
| 4 | KW10D | Grafiken sind möglichst komplex. |

**Instruktion**

| **Instruktion** |
| --- |
| Was trifft auf fachlich gute Gesundheits-Apps zu? |

**Items**

| **Nr.** | **Variablenname** | **Item** |
| --- | --- | --- |
| 1 | KW11A | Influencer werben für die App. |
| 2 | KW11B | Aussagen werden mit Quellen belegt. |
| 3 | KW11C | Autoren sind vom Fach und haben entsprechende Qualifikationen. |
| 4 | KW11D | Es gibt den Hinweis, dass die App den Arzt ersetzt. |

**Antwortoptionen**

| **Codierung** | **Antwortoption** |
| --- | --- |
| 0 | nicht ausgewählt |
| 1 | ausgewählt |
